# Structural equation modeling of direct and indirect associations of social media addiction with eating behavior in adolescents and young adults

**DOI:** 10.1038/s41598-023-29961-7

**Published:** 2023-02-21

**Authors:** Mohammad Ali Mohsenpour, Malihe Karamizadeh, Reza Barati-Boldaji, Gordon A. Ferns, Marzieh Akbarzadeh

**Affiliations:** 1 Nutrition Research Center, School of Nutrition and Food Sciences, Shiraz University of Medical Sciences, Shiraz, Iran; 2 Student Research Committee, Shiraz University of Medical Sciences, Shiraz, Iran; 3 Student Research Committee, Tabriz University of Medical Sciences, Tabriz, Iran; 4 Gastroenterohepatology Research Center, Shiraz University of Medical Sciences, Shiraz, Iran; 5 Division of Medical Education, Brighton and Sussex Medical School, Brighton, England, UK

**Keywords:** Nutrition, Nutrition disorders

## Abstract

Social media (SM) exerts important effects on health-related behaviors such as eating behaviors (EB). The present study was designed to determine the direct and indirect association of SM addiction with EB in adolescents and young adults through body image (BI). In this cross-sectional study, 12–22 years old adolescents and young adults, with no history of mental disorders or psychiatric medications usage were studied through an online questionnaire shared via SM platforms. Data were gathered about SM addiction, BI, and EB in its sub-scales. A single approach and multi-group path analyses were performed to find possible direct and indirect associations of SM addiction with EB through BI concerns. Overall, 970 subjects, 55.8% boys, were included in the analysis. Both multi-group (β = 0.484, SE = 0.025, P < 0.001) and fully-adjusted (β = 0.460, SE = 0.026, P < 0.001) path analyses showed higher SM addiction is related to disordered BI. Furthermore, the multi-group analysis showed one unit increment in SM addiction score was associated with 0.170 units higher scores for emotional eating (SE = 0.032, P < 0.001), 0.237 for external stimuli (SE = 0.032, P < 0.001), and 0.122 for restrained eating (SE = 0.031, P < 0.001). The present study revealed that SM addiction is associated with EB both directly and also indirectly through deteriorating BI in adolescents and young adults.

## Introduction

In recent years, communication through the internet has become an integral part of daily life^1^. In 2019, it has been reported that almost 72% of the world's population used online social networks, and this number is increasing every year^2^. In Iran, it was reported that in 2018, more than 47 million Iranians used online social networks^3^. Among internet-based social media users, adolescents are a large group that spends a prolonged time on these media. It has been reported that more than 88% of 13–18 years old adolescents in the United States have access to the internet. In 2018, 45% of American teenagers reported being almost constantly online, while this rate was 24% in 2015^4^. In Iran, it has been reported that in 2021, adolescents use smart devices for an average of 7.5 h a day^5^. Social media overuse was shown to be associated with several physical^6^ and mental health issues^7^. This excessive social media engagement can reach the point where it can be considered a form of addiction^8,9^.

The Internet and social media encourage users to create profiles with assigned profile pictures and it has been suggested that people's attractiveness in online profiles affects their popularity^10^. Hence, the online environment is full of images of peers and celebrities that create opportunities for social comparisons. Negative comparisons may occur when social media users compare their online images with others^11^. Body dissatisfaction which arises from these comparisons is known to be a major risk factor for body image concern and decreased self-esteem^12–14^. Pieces of evidences support the sociocultural theory which suggests social agents including the media, peers, and parents as determinants of internalized body shape ideals, and the most of time, these ideals are unrealistic ideals of body shape, thus leading to body image dissatisfaction^15,16^. Body image dissatisfaction can cause eating disorders such as bulimia nervosa and binge-eating disorder^17^ and lowering physical activity^18^. Therefore social media engagement or addiction might affect eating behaviors by increasing body image concerns.

Moreover, social media addiction was seen to affect behaviors toward health^19,20^ and eating^21^. Social media users are often exposed to dietary and health advices, which may have no scientific basis. Frequent sharing of food-related posts and advertisements on social media platforms may also lead to changes in eating behavior and an increase in the desire to eat unhealthy foods^1^.

In the case of adolescents, since a large part of the social and emotional development of this generation occurs on the internet and mobile phones, the impact of online social networks on them is much greater^22^. Most adolescents are at risk when using social media due to their limited ability for self-management and vulnerability to peer pressure^22^. While both genders are at risk, girls were shown to be more susceptible to social media risks^23^.

**Figure 1:**
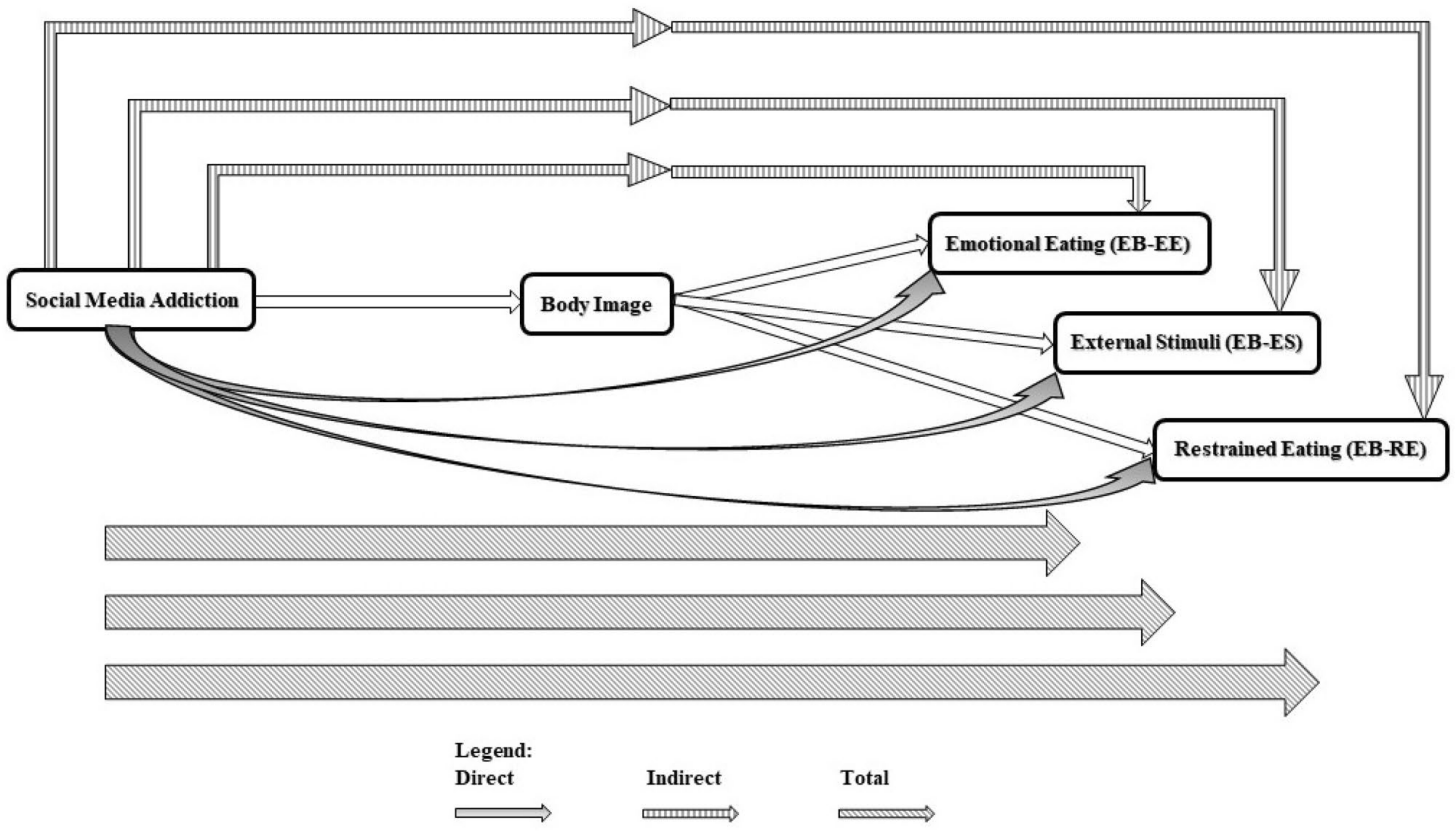
A schematic pathways of direct, indirect and total associations of multi-group path analysis between social media addiction, body image, and eating behavior.

Increasing the use of social media, especially among adolescents, has shown the need for various researches. Although several research projects were conducted on social media use and its effects on health-related behaviors in both western and non-western countries^4,6,20,24–27^, there are still information gaps in this area. Considering the important linkage between social media and health-related behaviors including eating habits both directly and indirectly through deteriorated body image, the present study was designed to determine the direct and indirect association of social media addiction with eating behavior in adolescents and young adults. We hypothesized that social media addiction can affect eating behaviors through body image deterioration. Figure 1 depicts a schematic pathways of direct, indirect, and total associations of multi-group path analysis between social media addiction, body image, and eating behavior.

## Methods

### Study design

A cross-sectional study was conducted from September 5, 2021, to October 2, 2021. The study was conducted among Iranian adolescents and young adults drawn from all regions in Iran. An online questionnaire was designed on the basis of the Porsline website (www.porsline.ir) to collect data. The link to the online questionnaire was sent through social media platforms (WhatsApp and Instagram) and the recipients were free to respond if they wished.

The survey questionnaire included a front-page describing the objectives, the target population, the details of the questions, and a statement that implied filling out the questionnaire is quite optional, and your voluntarily answering and finally submitting the questionnaire is considered as your consent to participate in the study. For teenagers, although they were allowed to have access to social media from their parents, there was also a statement to consult with their parents or legal guardians for participation. For this purpose, after the initial explanations on the front page, in the first question, we asked participants to state their consent (or in the case of teenagers for their legal guardian consent) by ticking the informed consent question as a requirement to enter the questionnaire. Thus, web-based informed consent was obtained from all subjects before participation. After initiating the answering to the questionnaire, participants were free to leave the survey at any stage before final submission, and the incomplete questionnaires were not saved. The protocol of the study was following the Declaration of Helsinki and was approved by the Institutional Review Board (IRB) and the Research Ethics Committee of Shiraz University of Medical Sciences (approval code: IR.SUMS.SCHEANUT.REC.1400.049). To prevent repeated responses, the domain restricted access using repeated IP addresses.

### Study population

Adolescents and young adults aged 12–22 years were eligible to participate in the study if they had access to devices that could connect to the internet and were literally skilled to be able to answer the questions on the form. Individuals with any history of diagnosed mental disorders or consuming any psychiatric medications were excluded from the study. The inclusion and exclusion criteria were mentioned on the front page of the online survey and were confirmed by the answers given to the related questions: “Have you ever had a history of mental disorders such as anxiety, depression, etc.?” and “If you have a history of mental disorders, have you ever received any medications?”.

Since the main goal of the present study was to conduct structural equation modeling and considering the fact that for this modeling, sample size estimation is not determined using closed-form formulas, rules of thumb were used for sample size determination. It is proposed to recruit 50–500 participants for this method^28^. To increase the study power and to ensure proper power for the multi-group analysis, we considered a larger sample size.

### Data gathering

The online survey comprised three main sections including the front page, the questionnaire, and the final closing page. After the front page with general information about the study, the online platform of the questionnaire showed each question on a single page. The questionnaire consisted of 4 main sections.

First, the demographic characteristics of participants such as age, gender (boy or girl), educational status (school student, college student, graduated, or leave), the highest educational level (high school, BS, MS, or general physician), following any diet (no special diet, weight gain, weight reduction, special medical diets, or sports diet), having network job (any online activity which demands spending time in social media to earn money), being the administrator of any social media pages (with the educational, entertainment, financial purposes, or not at all), and physical activity level (PAL) (sedentary, casual, moderate, or intense exercise)^29^ were recorded. The anthropometric records were self-reported for height and weight. Body mass index (BMI) was calculated using the reported data with the standard formula ([weight (kg)]/[height (m)]^2^). Socioeconomic status (SES) was recorded using the possession of 9 specific items by the household. Based on the number of items possessed by the household, participants were categorized into 3 groups deprived (possessed 3 items or less), semi-affluent (for possession of 4 to 6 items), and affluent (household possessed more than 6 items)^30^.

Three questions were used to identify ineligible participants. The first question was asked about age, thus records out of the predefined age range (12–22 years) were excluded. The second and third questions asked about any diagnosed mental disorders or consuming psychiatric medications. In case of positive answers to any of these questions, the respondent was removed from the final analysis.

In the second part, the dependence of individuals on social media was assessed using the Social Media Addiction Scale Student Form (SMAS-SF)^31^. This questionnaire was designed for students aged 12–22 years and its validity and reliability have been previously published^31^. This questionnaire consisted of 29 questions and 4 sub-dimensions with 5-point Likert-type responses including “1 = strongly disagree”, “2 = disagree”, “3 = neither agree nor disagree”, “4 = agree”, and “5 = strongly agree”. Sub-dimensions assessed respondents’ virtual tolerance, virtual communication, virtual problem, and virtual information. The scores ranged from 29 to 145. Higher scores indicate that the person considers him/herself more "social media addicted"^31^.

To validate the Persian version of the SMAS-SF based on the method of Lawshe^32^, first, the questionnaire was translated into Persian, and then back translation was done to confirm the concepts in Persian. The content validity was assessed by an expert panel composed of 10 specialists. The content validity ratio (CVR) and the content validity index (CVI) were evaluated. To test the reliability, a test–retest was done. The questionnaire was completed by 40 participants in the age range of 12–25 years old twice with a 3 weeks intervals.

In the third part, the Dutch Eating Behavior Questionnaire (DEBQ) was used to evaluate respondents’ eating behavior^33^. The validity and reliability of the Persian version of this questionnaire were previously confirmed by Kargar et al.^34^. The questionnaire contained 33 questions in 3 sub-domains. The first sub-domain includes questions related to emotional eating (EB-EE) indicating overeating in response to emotions, which includes 13 questions. The second sub-domain consists of 10 questions and measures eating in response to external stimuli (EB-ES) (food-related stimuli, regardless of states of hunger and satiety). The third sub-domain contains 10 questions and evaluates restrained eating (EB-RE) which assesses trying to avoid eating. The questions are ranked on a 5-point Likert scale scored as “0 = never”, “1 = seldom”, “2 = sometimes”, “3 = often”, and “4 = frequently”. In the sub-domains of DEBQ, the increments in the score from 0 to the maximum score means as form not eating in response to emotions to overeating due to an emotional state such as nervousness, happiness, or excitement for EB-EE, not paying any attention to these stimuli and only eat when they are really hungry to eating in response to stimuli such as color, smell and taste of food for EB-ES, and not avoid eating to avoid eating in EB-RE^34^. To determine the participant’s score for each sub-domain, the mean score for the sub-domain was calculated using the sum of scores earned for questions divided by the number of questions in the sub-domain.

In the last section of the online form, the information related to body image concerns was evaluated by the Body Image Concern Inventory (BICI)^35^. The validity and reliability of the Persian version of this questionnaire were previously confirmed by Pooravari et al.^36^. This questionnaire is a 5-point Likert type that contains 19 questions with the answers and scores of “never = 1”, “seldom = 2”, “sometimes = 3”, “often = 4”, and “frequently = 5”. The total score of the questionnaire varies from 19 to 95. A score of 19–37 indicates none or very low concern about body image, a score of 38–52 indicates low concern about body image, a score of 53–69 indicates moderate concern about body image, and a score of 70 and above indicates a high concern of body image^36^. The BICI also assesses body image in two sub-domain dissatisfaction and embarrassment of one's appearance, checking and hiding perceived defects (BI—dissatisfaction), and the degree to which anxiety about appearance interferes with a person's social performance (BI—social performance).

### Statistical analyses

The normality of the data was assessed using the Kolmogorov–Smirnov test. Quantitative data with normal distribution were reported as mean and standard deviation (SD) and were compared using an independent sample t-test between genders. For the independent sample t-test, t (df) and effect size (Cohen’s d) were reported. Cohen’s d value is interpreted as small, medium, and large effect size considering 0.2, 0.5, and 0.8 thresholds^37,38^. Qualitative variables were reported as frequency and percentage and were analyzed using the chi-square or Fisher’s exact test in the case of cells with expected counts less than 5^39^. For the chi-square test, χ^2^ (df), and for Fisher’s exact test, Fisher’s value were reported. For 2 × 2 categorical variable analysis, φ statistics, and for analyzing variables with more than two categories, Cramer’s V were reported^39^.

Internal consistency of each scale and sub-domains of each questionnaire were assessed in the study population using Cronbach’s α test. Cronbach’s α > 0.7 is considered an acceptable internal consistency^40^. Statistical analyses were performed using SPSS software version 20. Mplus software (version 6) was used to run a path analysis which is a subset of structural equation modeling. A single approach path analysis with a bootstrap approach was run in order to assess the direct and indirect associations of social media addiction with eating behavior through body image concern in the total population. We conducted a multi-group path analysis investigating direct and indirect associations between social media addiction, EB-EE, EB-ES, and EB-RE through BI. To evaluate gender differences we repeated multi-group path analysis with an assumption of equality in coefficient in both genders. The model was properly fitted to data based on the model fit criteria proving that our assumption of equality was valid. Gender, age, network job, being the admin of any social media pages, PAL, BMI, and SES were entered in the multivariate model as potential confounders. To indicate any linkages between variables of interest, path coefficients were reported which are standardized versions of linear regression weights in the structural equation modeling approach. The root means square error of approximation (RMSEA), comparative fit index (CFI), and Tucker–Lewis index (TLI) were calculated to check the model fit. The values for CFI and TLI > 0.95 and RMSEA < 0.06 are considered to be acceptable^41^. According to the well-defined clinical correlation between study variables including AD, BI, and EB subclasses, the path analysis was conducted as a full model and all pairwise correlations were considered. Therefore, reporting model fit criteria were not necessary for fully fitted models^42^. A P value less than 0.05 was considered statistically significant.

## Results

During the time that the link of the online questionnaire was active, 1492 unique IPs viewed the questionnaire description (the front page), and of these, 1214 individuals completed the questionnaire which corresponds to an 81.37% response rate. Based on the answer to the question on the use of psychiatric medications, 244 respondents were excluded, 78 individuals did not answer the question and 166 stated using these medications. Finally, 970 subjects (44.2% girls and 55.8% boys) were included in the analysis. The mean age of the included participants was 17.99 ± 2.53 years (18.34 ± 2.42 and 17.55 ± 2.60 years for boys and girls, respectively, t (887.75) = 4.80, Cohen’s d = 0.31 [medium effect], P < 0.001). BMI of the participants were 22.61 ± 5.05 kg/m^2^ (22.95 ± 5.24 and 22.18 ± 4.74 kg/m^2^ for boys and girls respectively, t (968) = 2.36, Cohen’s d = 0.15 [small effect], P = 0.017). Students were the most popular (46.3% for high school and 42.7 for college student) respondents in the study. Regarding diet, 79.2% of the participants reported no special diet, and others reported weight reduction (9.9%), weight gain (3.1%), medical diets (0.2%), and sports diets (7.6%). Boys and girls were unequally distributed in diets (Fisher’s value = 11.90, Cramer’s V = 0.10, P = 0.012). A sedentary lifestyle was declared by 54.0% of the participants, while casual, moderate, and intense exercise was respectively reported by 24.6%, 13.4%, and 7.9%. The distribution of boys and girls in PAL was also significantly different (χ^2^ (3) = 40.54, Cramer’s V = 0.20, P < 0.001) (Table 1).

**Table 1:** Demographic characteristics of total population as well as genders.

	Total (n = 970)	Boys (n = 541)	Girls (n = 429)	t (df), effect size *	P value
	Mean ± SD	Mean ± SD	Mean ± SD		
Age (year)	17.99 ± 2.53	18.34 ± 2.42	17.55 ± 2.60	4.80 (887.75), 0.31	< 0.001^‡^
Height (cm)	171.21 ± 10.70	177.41 ± 8.85	163.40 ± 7.11	27.31 (968.82), 1.74	< 0.001^‡^
Weight (kg)	66.68 ± 17.74	72.53 ± 18.32	59.30 ± 13.82	12.81 (965.68), 0.81	< 0.001^‡^
BMI (kg/m^2^)	22.61 ± 5.05	22.95 ± 5.24	22.18 ± 4.77	2.36 (968), 0.15	0.018^‡^

	N (%)	N (%)	N (%)	χ^2^ (df), φ/Cramer’s V	P value
Education status				11.740 (3), 0.11^#^	**0.008**^§^
School student	449 (46.3)	232 (42.9)	217 (50.6)		
College student	414 (42.7)	235 (43.4)	179 (41.7)		
Graduated	59 (6.1)	43 (7.9)	16 (3.7)		
Leave	48 (4.9)	31 (5.7)	17 (4.0)		
Education level				4.38, 0.07^#^	0.217^¶^
High school	607 (62.6)	333 (61.6)	274 (63.9)		
BSc	347 (35.8)	199 (36.8)	148 (34.5)		
MSc or higher	12 (1.2)	5 (0.9)	7 (1.6)		
MD or higher	4 (0.4)	4 (0.7)	0		
SES				1.12 (2), 0.03^#^	0.569^§^
Deprived	238 (24.5)	128 (23.7)	110 (25.6)		
Semi-affluent	424 (43.7)	234 (43.3)	190 (44.3)		
Affluent	308 (31.8)	179 (33.1)	129 (30.1)		
Diet				11.90, 0.10^#^	**0.012**^¶^
No	768 (79.2)	427 ( (78.9)	341 (79.5)		
Yes—weight reduction	96 (9.9)	44 (8.1)	52 (12.1)		
Yes—weight gain	30 (3.1)	16 (3.0)	14 (3.3)		
Yes—medical diet	2 (0.2)	1 (0.2)	1 (0.2)		
Yes—sport	74 (7.6)	53 (9.8)	21 (4.9)		
Network job				2.69 (1), − 0.05^†^	0.101^§^
No	891 (91.9)	490 (90.6)	401 (93.5)		
Yes	79 (8.1)	51 (9.4)	28 (6.5)		
Admin				1.24 (3), 0.03^#^	0.743^§^
No	801 (82.6)	443 (81.9)	358 (83.4)		
Yes—educational	30 (3.1)	16 (3.0)	14 (3.3)		
Yes—job	44 (4.5)	24 (4.4)	20 (4.7)		
Yes—fun	95 (9.8)	58 (10.7)	37 (8.6)		
PAL				40.54 (3), 0.20^#^	** < 0.001**^§^
Sedentary	524 (54.0)	250 (46.2)	274 (63.9)		
Casual exercise	239 (24.6)	140 (25.9)	99 (23.1)		
Moderate exercise	130 (13.4)	91 (16.8)	39 (9.1)		
Intense exercise	77 (7.9)	60 (11.1)	17 (4.0)		

P < 0.05 considered significant. Significant results are shown in bold font.

*BMI* body mass index, *SES* socioeconomic status, *PAL* physical activity level.

*Cohen’s d, interpreted as small (0.2), medium (0.5), and large (0.8) effects.

^#^Cramer’s V.

^†^φ statistics.

^‡^Independent sample t-test.

^§^Chi-square test.

^¶^Fisher’s exact test.

The CVR for all questions in the Persian version of the SMAS-SF was above 80%. The CVI for relevancy was > 80%, CVI for clarity was > 70% and the CVI for simplicity was > 80% for all questions. The Pearson correlation coefficient was 0.87 (P value < 0.001) between the results of 3 weeks of test–retest. Cronbach's α was yielded to be 0.92 for the overall SMAS-SF. The sub-domains Cronbach’s α resulted in acceptable internal consistency (virtual tolerance: 0.80, virtual communication: 0.83, virtual problem: 0.86, and virtual information: 0.76).

The analysis showed desirable internal consistency of the BICI questionnaire (Cronbach's α for total = 0.94, BI-dissatisfaction = 0.90, and BI-social performance = 0.88). In the sub-domains of DEBQ Cronbach’s α was 0.88, 0.91, and 0.79, for EB-EE, EB-ES, and EB-RE, respectively.

Table 2 summarizes the social media addiction, body image, and eating behavior status of the total population as well as scores among genders. Total scores for social media addiction was 82.83 ± 19.99 and it was significantly higher in girls compared to boys (t (968) = − 3.43, Cohen’s d = 0.22 [medium effect], P = 0.001). Besides girls had significantly higher scores in subclasses of social media addiction such as virtual tolerance (t (877.96) = − 4.06, Cohen’s d = 0.26 [medium effect], P < 0.001), virtual communication (t (968) = − 3.96, Cohen’s d = 0.25 [medium effect], P < 0.001) and virtual problem (t (968) = − 2.67, Cohen’s d = 0.17 [small effect], P = 0.008). The body image concern score was 86.56 ± 17.92. Compared to boys, girls had higher scores in the subscale of BI-dissatisfaction (t (881.35) = − 2.10, Cohen’s d = 0.13 [small effect], P = 0.035). Also, 37.1% of the participants reported none or very low body image concern and 12.5% of the participants reported a high level of body image concern. The mean scores of emotional eating, external eating, and restrained eating were 2.44 ± 0.90, 3.24 ± 0.74, and 2.30 ± 0.94, respectively, and there were no significant differences between the two sexes.

**Table 2:** The social media addiction, body image concern, and eating behavior status of total population as well as scores among genders.

	Total (n = 970)	Boys (n = 541)	Girls (n = 429)	t (df), effect size^†^	P value
	Mean ± SD	Mean ± SD	Mean ± SD		
Addiction total	82.83 ± 19.99	80.87 ± 19.24	85.29 ± 20.66	− 3.43 (968), 0.22	0.001*
Virtual tolerance	16.15 ± 4.35	15.65 ± 4.14	16.79 ± 4.53	− 4.06 (877.96), 0.26	< 0.001*
Virtual communication	25.77 ± 6.99	24.98 ± 6.72	26.76 ± 7.20	− 3.96 (968), 0.25	< 0.001*
Virtual problem	23.62 ± 7.37	23.06 ± 7.26	24.33 ± 7.45	− 2.67 (968), 0.17	0.008*
Virtual information	17.27 ± 4.78	17.17 ± 4.92	17.39 ± 4.59	− 0.70 (968), 0.04	0.476*
BI concern score	46.56 ± 17.92	45.65 ± 17.09	47.71 ± 18.88	− 1.75 (872.89), 0.11	0.079*
BI—dissatisfaction	24.39 ± 10.45	23.75 ± 10.04	25.19 ± 10.90	− 2.10 (881.35), 0.13	0.035*
BI—social performance	22.16 ± 8.21	21.89 ± 7.92	22.51 ± 8.56	− 1.16 (883.19), 0.07	0.246*
EB-EE	2.44 ± 0.90	2.41 ± 0.88	2.48 ± 0.92	− 1.14 (968), 0.07	0.251*
EB-ES	3.24 ± 0.74	3.24 ± 0.75	3.25 ± 0.72	− 0.14 (968), 0.01	0.887*
EB-RE	2.30 ± 0.94	2.28 ± 0.91	2.34 ± 0.96	− 0.92 (968), 0.06	0.357*

	N (%)	N (%)	N (%)	χ^2^ (df), Cramer’s V	P value
BI [n (%)]				3.56 (3), 0.06	0.312^#^
None or very low	360 (37.1)	201 (37.2)	159 (37.1)		
Low	267 (24.5)	159 (29.4)	108 (25.2)		
Moderate	222 (22.9)	121 (22.4)	101 (23.5)		
High	121 (12.5)	60 (11.1)	61 (14.2)		

P < 0.05 considered significant. Significant results are shown in bold font.

*BI* body image concern, *EB-EE* Eating behavior-Emotional eating, *EB-ES* Eating behavior-External stimuli, *EB-RE* Eating behavior-Restrained eating.

*Independent sample t-test.

^#^Chi-square test.

^†^Cohen’s d, interpreted as small (0.2), medium (0.5), and large (0.8) effects.

Based on the results of path analysis, each unit increment in social media addiction score was associated with 0.484 units (SE = 0.025, P < 0.001) higher score of body image concern.

**Figure 2:**
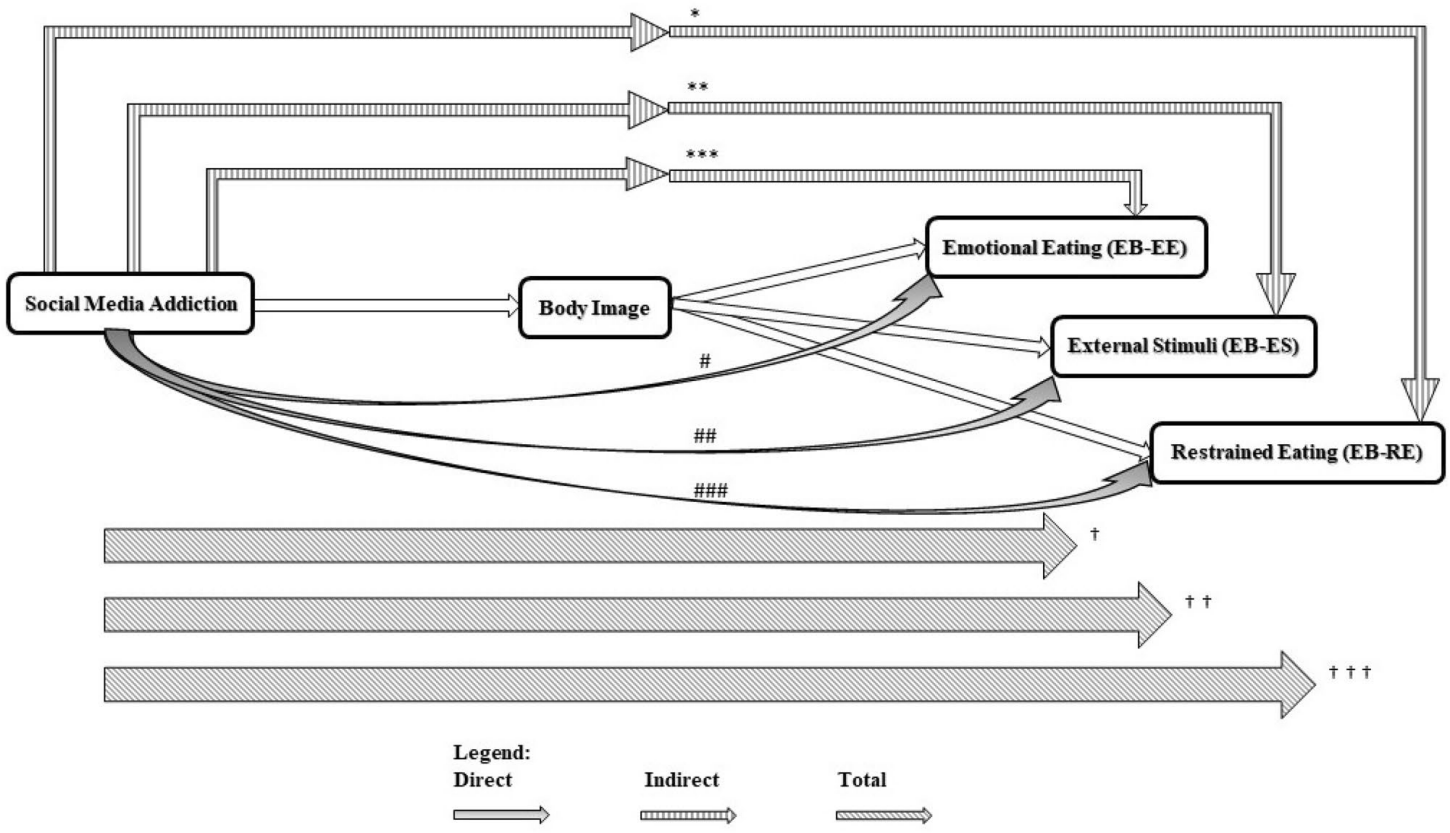
Multi-group path analysis of direct, indirect and total associations between social media addiction, body image, and eating behavior. ^*^β = 0.069, P < 0.001; ^**^β = 0.065, P < 0.001; ^***^β = 0.136, P < 0.001; ^#^β = 0.102, P = 0.004; ^##^β = 0.172, P < 0.001; ^###^β = − 0.014, P = 0.687; ^†^β = 0.170, P < 0.001; ^††^β = 0.237, P < 0.001; ^†††^β = 0.122, P < 0.001.

Table 3 shows the multi-group direct and indirect associations of social media addiction and the body image score with eating behavior. The crude multi-group analysis indicates that social media addiction was significantly related to deteriorated eating behavior both directly and indirectly, except direct link with restrained eating which was not statistically significant (β = − 0.014, SE = 0.035, P = 0.687). Besides, the body image concern showed a statistically significant association with eating behavior. The direct, indirect, and total associations of multi-group paths analysis between social media addiction, body image, and eating behavior is depict in Fig. 2.

**Table 3:** The associations of social media addiction with participants’ body image concern and eating behavior: a multi-group path analysis model.

	EB-EE			EB-ES			EB-RE		
	β	SE	P	β	SE	P	β	SE	P
Social media addiction									
Direct	0.102	0.035	**0.004**	0.172	0.035	** < 0.001**	− 0.014	0.035	0.687
Indirect	0.069	0.017	** < 0.001**	0.065	0.016	** < 0.001**	0.136	0.019	** < 0.001**
Total	0.170	0.032	** < 0.001**	0.237	0.032	** < 0.001**	0.122	0.031	** < 0.001**
Body image concern	0.142	0.035	** < 0.001**	0.135	0.032	** < 0.001**	0.281	0.034	** < 0.001**

P value for path-analysis. P < 0.05 considered significant. Significant results are shown in bold font.

*EB-EE* Eating behavior-Emotional eating, *EB-ES* Eating behavior-External stimuli, *EB-RE* Eating behavior-Restrained eating.

Table 4 shows the multi-group direct and indirect associations of social media addiction and the body image score with eating behaviors’ based on gender. Considering the equality in coefficients for genders, the model was properly fitted to data (RMSEA = 0.030, CFI = 0.995, and TLI = 0.968). Based on the results of path analysis, each unit increment in social media addiction score was associated with a higher score of body image concern (β = 0.433, SE = 0.025, P < 0.001). In both genders, social media addiction was associated with worsened eating behavior both directly and indirectly through body image. The result was not significant for the direct association of social media addiction on EB-RE (β = − 0.001, SE = 0.002, P = 0.691, for both genders).

**Table 4:** The associations of social media addiction with participants’ body image concern and eating behaviors’ based on gender: a multi-group path analysis model.

	EB-EE			EB-ES			EB-RE		
	β	SE	P	β	SE	P	β	SE	P
Boys									
Social media addiction									
Direct	0.004	0.002	**0.005**	0.006	0.001	** < 0.001**	- 0.001	0.002	0.691
Indirect	0.003	0.001	** < 0.001**	0.002	0.001	** < 0.001**	0.006	0.001	** < 0.001**
Total	0.008	0.002	** < 0.001**	0.009	0.001	** < 0.001**	0.006	0.002	** < 0.001**
Body Image concern	0.007	0.002	** < 0.001**	0.006	0.001	** < 0.001**	0.015	0.002	** < 0.001**
Girls									
Social media addiction									
Direct	0.004	0.002	**0.005**	0.006	0.001	** < 0.001**	- 0.001	0.002	0.691
Indirect	0.003	0.001	** < 0.001**	0.002	0.001	** < 0.001**	0.006	0.001	** < 0.001**
Total	0.008	0.002	** < 0.001**	0.009	0.001	** < 0.001**	0.006	0.002	** < 0.001**
Body Image concern	0.007	0.002	** < 0.001**	0.006	0.001	** < 0.001**	0.015	0.002	** < 0.001**

The models (associations of social media addiction and body image concern with eating behavior) were properly fitted for our data (RMSEA = 0.030, CFI = 0.995, and TLI = 0.986, for both models based on gender).

P value for path-analysis. P < 0.05 considered significant. Significant results are shown in bold font.

*EB-EE* Eating behavior-Emotional eating, *EB-ES* Eating behavior-External stimuli, *EB-RE* Eating behavior-Restrained eating.

Table 5 indicates the direct associations of variables with body image and eating behavior. Social media addiction (β = 0.460, SE = 0.026, P < 0.001) and BMI (β = 0.216, SE = 0.028, P < 0.001) were associated with body image scores. EB-EE was related to social media addiction (β = 0.123, SE = 0.036, P = 0.001), body image (β = 0.132, SE = 0.036, P < 0.001), having network job (β_yes/no_ = 0.075, SE = 0.032, P = 0.019), and BMI (β = 0.067, SE = 0.033, P = 0.040). Social media addiction (β = 0.167, SE = 0.036, P < 0.001), body image (β = 0.153, SE = 0.036, P < 0.001), BMI (β = − 0.073, SE = 0.033, P = 0.025) and being semi-affluence compared to deprived (β = 0.115, SE = 0.039, P = 0.003) were recognized as associated variables with EB-ES. Body image (β = 0.241, SE = 0.033, P < 0.001), age (β = − 0.080, SE = 0.030, P = 0.008), BMI (β = 0.275, SE = 0.030, P < 0.001), and physical activity (β = 0.125, SE = 0.030, P < 0.001) and being semi-affluence compared to deprived (β = − 0.098, SE = 0.036, P = 0.007) associated with EB-RE. One unit increment in social media addiction score was associated with a 0.184 unit higher score for emotional eating (SE = 0.032, P < 0.001) [β = 0.123, SE = 0.036, P = 0.001 for direct and β = 0.061, SE = 0.017, P < 0.001 for indirect effects], 0.237 units for external eating (SE = 0.031, P < 0.001) [β = 0.167, SE = 0.036, P < 0.001 for direct and β = 0.070, SE = 0.017, P < 0.001 for indirect effects], and 0.140 units for restrained eating (SE = 0.031, P < 0.001) [β = 0.029, SE = 0.034, P = 0.390 for direct and β = 0.111, SE = 0.017, P < 0.001 for indirect effects].

**Table 5:** Direct associations of variable with body image concern and eating behavior.

	Body Image concern			EB-EE			EB-ES			EB-RE		
	β	SE	P	β	SE	P	β	SE	P	β	SE	P
Social Media Addiction	0.460	0.026	** < 0.001**	0.123	0.036	**0.001**	0.167	0.036	** < 0.001**	0.029	0.034	0.390
Body Image concern	–	–	–	0.132	0.036	** < 0.001**	0.153	0.036	** < 0.001**	0.241	0.033	** < 0.001**
Gender (male)	− 0.024	0.029	0.405	− 0.029	0.032	0.372	0.024	0.032	0.445	− 0.047	0.030	0.114
Age (year)	− 0.026	0.029	0.363	− 0.040	0.032	0.215	0.024	0.032	0.452	− 0.080	0.030	**0.008**
Network Job (yes)	0.019	0.028	0.506	0.075	0.032	**0.019**	0.025	0.032	0.432	0.052	0.030	0.080
Admin (yes)	− 0.028	0.028	0.329	0.033	0.032	0.297	− 0.016	0.032	0.609	0.029	0.030	0.339
PAL (sedentary)	0.026	0.029	0.358	0.048	0.032	0.139	− 0.009	0.032	0.774	0.125	0.030	** < 0.001**
BMI (kg/m^2^)	0.216	0.028	** < 0.001**	0.067	0.033	**0.040**	− 0.073	0.033	**0.025**	0.275	0.030	** < 0.001**
Semi-affluence^#^	− 0.017	0.035	0.624	0.032	0.039	0.417	0.115	0.039	**0.003**	− 0.098	0.036	**0.007**
Affluence^#^	− 0.008	0.035	0.822	0.042	0.039	0.281	0.051	0.039	0.186	< 0.001	0.036	0.992

P value for path-analysis. P < 0.05 considered significant. Significant results are shown in bold font.

*EB-EE* Eating behavior-Emotional eating, *EB-ES* Eating behavior-External stimuli, *EB-RE* Eating behavior-Restrained eating, *PAL* Physical activity level, *BMI* body mass index.

*Positive answer compared to negative.

^#^Compared to deprived.

## Discussion

Social networking platforms have become the main routes of communication in most countries. This means of communication has several advantages but also has potential threats^13^. The present cross-sectional study determined the direct and indirect associations of social media addiction with eating behavior in adolescents and young adults. Based on the study results, both in the crude and adjusted models, social media addiction was significantly associated with higher levels of body image concern, and body image concern was significantly associated with a deteriorated eating behavior in all three subscales of emotional eating, external stimuli, and restrained eating. Moreover, social media addiction was associated with higher scores of emotional eating and external stimuli both directly and indirectly. Multi-group analysis considering genders showed similar results for boys and girls. After adjustment for potential confounders, a similar result was seen. On the other hand, social media addiction did not have significant direct associations with restrained eating, both in the crude and adjusted models. But it was significantly associated with increased scores of restrained eating indirectly through body image concerns.

Adolescence is the most common period for body image concerns^15^. Body image dissatisfaction especially arises when a teenager compares his or her appearance to a goal that is unattainable and observes a discrepancy with others^13^. It was previously shown that regular use of highly-visual social media (HVSM) for more than 2 h a day was associated with increased body image concerns in adolescents, in comparison to those with no involvement in HVSM^16^. In the present study, it was revealed that social media addiction was significantly associated with increased body image concerns in adolescents and young adults. Also, a study by Ho et al.^43^ revealed that social media comparison with friends and celebrities is associated with adolescents’ body image dissatisfaction and drives them to be thin and muscular in both genders^43^. When the assessment of individuals’ body image differs from other pictures on social media, individuals are more likely to feel dissatisfied and concerned about their appearance^16^. A sense of body dissatisfaction prompts individuals to adjust their attitudes and behaviors such as eating behaviors in order to reduce the reality gap^44^.

The determinants of eating habits are highly complex, especially in adolescents^45^, including parental care and behavior^46,47^, peer pressure^46^, food exposure^46^, food commercials on media^46^, food cost^48^, food availability^48^, negative self-evaluation^45,47^, body dissatisfaction^45,49^, internalization of the thin beauty ideal^49^, and social media engagement^50^. From these factors, body dissatisfaction was suggested as an independent predictor of eating disorders^51^. In the present study, it was shown that body image concern was significantly associated with deteriorated eating behavior in all three subscales of emotional eating, external stimuli, and restrained eating.

Body image dissatisfaction is believed to deteriorate eating behaviors by means of factors including negative emotional status, female gender, peer influence, and early puberty among girls, social and familial factors. These factors lead person to believe being overweight or obese. Thus, despite having a normal weight, person tries to obey weight-reduction diet, and in some cases extreme diets, that consequently initiate eating disorders^52^. The contents of social media are often a source of comparison for both boys and girls^16^.

The pooled results of a meta-analysis of 22 studies examining the associations between social media with body image concern and eating habits by Zhang et al.^2^ revealed a weak but significant positive association between the usage of social network engagement and body image concern as well as disordered eating behavior^2^. Besides Aparicio-Martinez et al.^53^ showed that addiction to social network sites is associated with unhealthy eating habits, a thinner body image, and a desire to change part of the body. Acar et al. in 2020^54^, investigated the role of physical appearance comparison in daily life and on social media on eating behaviors in 1384 adolescents. According to their results, more immersion in social media can lead to more physical appearance comparison and disordered eating behaviors in daily life^54^. In a similar way, Rodgers et al. in 2020^14^ showed the effects of social media engagement on appearance comparison, body dissatisfaction, eating disorders, lower self-esteem, and higher depression among adolescents^14^. According to their study, appearance comparisons, internalization of the social media ideal, and the muscular ideal appeared as the strongest factor associated with body image concern and restrained eating^14^. To summarize, as was shown in the present study, social media addiction can be associated with eating habits indirectly through deteriorating body image.

Besides deteriorating eating behavior secondary to increased body image concerns, social media might lead to unhealthy eating habits through different behavioral changes. For example, omitting breakfast in social media users is popular probably through the displacement of other activities. Overspending time on social network sites can result in a lack of time for eating breakfast and an alteration of circadian rhythmicity towards a later midpoint of sleep and subsequent breakfast skipping^24^. Engagement in social media can disturb the duration and quality of sleep^55^. Sleep deprivation is associated with insulin resistance, increased hunger, and decreased satiety resulting in unhealthy dietary patterns^56^. Moreover, social media can affect eating behavior through food advertisements. Social media provide an attractive environment for food marketers to publicize their products, and food advertising plays a significant role in food choices and preferences among young adults^24^. Results of the present study revealed that social media addiction was associated with increased scores of emotional eating and external stimuli both directly and indirectly. After adjustment for potential confounders, a similar result was seen. It should be noted that in both models, direct relations were more pronounced compared to the indirect association.

The present study had several limitations and strong points. Our cross-sectional design was not able to establish causality. The study was conducted during the Covid-19 pandemic and lockdowns. It was seen that lockdowns might lead to mental health issues and consequently change the eating habits of the affected individuals^57^. Moreover, findings for the effect of psychiatric medication on eating behavior are contradicted^58^. Thus, to avoid bias in our results and generalizability, we excluded individuals with any history of diagnosed mental disorders or consuming any psychiatric medications, which can limit our findings. In addition, as a limitation of the present study, online data collection may have led to enhanced results in social media addiction scores, since the questionnaire was distributed through social media and the possibility of answering the questionnaire was higher in those who were more dependent on social media. Besides, data on weight and height were self-reported and it might be prone to under/over-reporting biases. Due to the limitations related to the online survey, we had to reduce the number of questions as much as possible, therefore we assessed physical activity level only through one question. Although the online survey led to some limitations, it helped us to include nationwide participants. Moreover, it might have led to more precise answers to social media addiction and body image concern questions. Social media engagement might be in part related to educational or career-related purposes, but the questionnaire used in the present study did not consider those activities. To the best of our knowledge, the present study was the first to assess the direct and indirect associations of social media addiction on eating behavior through body image concerns.

It is suggested in future studies, the specific content that individuals are exposed in social media be assessed to see how this affects eating behavior. The time spent on social media may be worth assessment. Also, it is suggested that the concurrent effects of nutritional knowledge and food-related social media contents be assessed on eating behavior.

## Conclusion

The present study mainly revealed that social media addiction might be associated with eating behavior both directly and also indirectly through deteriorating body image in adolescents and young adults. It is suggested future studies are needed to assess these associations considering the types of social media and the contents participants are faced with through social media.

## Data Availability

The datasets used and/or analyzed during the current study are available from the corresponding author on reasonable request.

## Abbreviations

SE: Standard error
IRB: Institutional Review Board
BS: Bachelor of Science
MS: Master of Science
PAL: Physical activity level
BMI: Body mass index
SES: Socio-economic status
SMAS-SF: Social Media Addiction Scale-Student Form
CVR: Content validity ratio
CVI: Content validity index
DEBQ: Dutch Eating Behavior Questionnaire
EB-EE: Eating behavior-emotional eating
EB-ES: Eating behavior-external stimuli
EB-RE: Eating behavior-restrained eating
BICI: Body image concern inventory
BI: Body image
RMSEA: Root mean square error of approximation
CFI: Comparative fit index
TLI: Tucker–Lewis index
HVSM: Highly-visual social media
SD: Standard deviation
